# Supplementary material for: MicroRNAs Let-7b-5p and miR-24-3p as Potential Therapeutic Agents Targeting Pancreatic Cancer Stem Cells
Source: Int J Mol Sci. 2025 Nov 15;26(22):11066. doi: 10.3390/ijms262211066 (PMC12652030; doi:10.3390/ijms262211066)
Supplement: Supplementary file 1 [file ijms-26-11066-s001.zip › Supplementary figures plus legends151025.pdf]

hsa-Let-7b-5p

74-84 of STAT3 3´UTR	5´................GCGCUACCUCC
hsa-Let-7b-5p	3´-UUGGUGUGUUGGAUGAUGGAGU
99-105 of IGF1R 3´UTR	5´..............................UCUUACCUCA
hsa-Let-7b-5p	3´-UUGGUGUGUUGGAUGAUGGAGU
274-280 of SOCS1 3´UTR	5´..........................UUCCUACCUCU
hsa-Let-7b-5p	3´-UUGGUGUGUUGGAUGAUGGAGU

hsa-Let-7a-5p

227-233 of WNT1 3´ UTR	5´..................GACCUACCUCC
hsa-Let-7a-5p	3´-UUGAUAUGUUGGAUGAUGGAGU
2500-2506 of GPR63 3´ UTR	5´..................CACCUACCUCU
hsa-Let-7a-5p	3´-UUGAUAUGUUGGAUGAUGGAGU
1894-1900 of MYC 3´ UTR	5´..................UAAUUACCUCA
hsa-Let-7a-5p	3´-UUGAUAUGUUGGAUGAUGGAGU

hsa-miR-23a-3p

1637-1643 of SMAD 3´ UTR	5´.......................UCAAAUGUGAU
hsa-miR-23a-3p	3´-CCUUUAGGGACCGUUACACUA
426-433 of FZD5 3´ UTR	5´.......................AAAAAAUGUGA..
hsa-miR-23a-3p	3´-CCUUUAGGGACCGUUACACUA
1699-1704 of MYC exon 3	5´.......................UGUAAUGUGCU
hsa-miR-23a-3p	3´-CCUUUAGGGACCGUUACACUA

hsa-miR-191-5p

77-83 of FZD5 3´ UTR	5´.......................CACUUCCGUUU
hsa-miR-191-5p	3´-GUCGACGAAAACCCUAAGGCAAC
813-820 of TMC7 3´ UTR	5´.......................GAUUUCCGUUA
hsa-miR-191-5p	3´-GUCGACGAAAACCCUAAGGCAAC
186-193 of TAF5 3´ UTR	5´.......................AAUUUCCGUUA
hsa-miR-191-5p	3´-GUCGACGAAAACCCUAAGGCAAC

hsa-Let-7c-5p

77-83 of ACVR1B 3´ UTR	5´..................GCCCUACCUCU
hsa-Let-7c-5p	3´-UUGGUAUGUUGGAUGAUGGAGU
137-143 of RPUSD2 3´ UTR	5´..................UUGCUACCUCC
hsa-Let-7c-5p	3´-UUGGUAUGUUGGAUGAUGGAGU
1894-1900 of MYC 3´ UTR	5´....................UAAUUACCUCA
hsa-Let-7c-5p	3´-UUGGUAUGUUGGAUGAUGGAGU

hsa-miR-24-3p

2700-2706 of DVL3 3´ UTR	5´.........................UCUCUGAGCCU
hsa-miR-24-3p	3´-GACAAGGACGACUUGACUCGGU
3859-3866 of FZD5 3´ UTR	5´.........................UUACUGAGCCA
hsa-miR-24-3p	3´-GACAAGGACGACUUGACUCGGU
2355-2361 of WNT2 3´ UTR	5´.........................UGAUGAGCCAA
hsa-miR-24-3p	3´-GACAAGGACGACUUGACUCGGU

hsa-Let-7e-5p

274-280 of SOCS1 3´ UTR	5´..................CUCCUACCUCU
hsa-Let-7e-5p	3´-UUGAUAUGUUGGAGGAUGGAGU
1210-1217 of FNIP1 3´ UTR	5´..................CUACUACCUCA
hsa-Let-7e-5p	3´-UUGAUAUGUUGGAGGAUGGAGU
1894-1900 of MYC 3´ UTR	5´..................UAAUUACCUCA
hsa-Let-7e-5p	3´-UUGAUAUGUUGGAGGAUGGAGU

hsa-miR-23b-3p

1001-1008 of GSK3β 3´ UTR	5´.......................AAUAAUGUGAA
hsa-miR-23b-3p	3´-CCAUUAGGGACCGUUACACUA
59-65 of ACVR1C 3´ UTR	5´.......................CCUUAUGUGAA
hsa-miR-23b-3p	3´-CCAUUAGGGACCGUUACACUA
5252-5258 of ACVR1C 3´ UTR	5´.......................CCUAAUGUGAU
hsa-miR-23b-3p	3´-CCAUUAGGGACCGUUACACUA

hsa-miR-103a-3p

810-816 of REST 3´ UTR	5´..........................CUUUGCUGCA
hsa-miR-103a-3p	3´-AGUAUCGGGACAUGUUACGACGA
1372-1379 of WNT16 3´ UTR	5´.........................GUAAUGCUGCA
hsa-miR-103a-3p	3´-AGUAUCGGGACAUGUUACGACGA
412-418 of AXIN 3´ UTR	5´.........................GAUAUGCUGCU
hsa-miR-103a-3p	3´-AGUAUCGGGACAUGUUACGACGA

hsa-miR-1246

510-517 of GSK3β 3´ UTR	5´.......................CCUAAUCCAUA
hsa-miR-1246	3´-GGACGAGGUUUUUAGGUAA
89-95 of ACVR1 3´ UTR	5´.......................UGGAAUCCAUC
hsa-miR-1246	3´-GGACGAGGUUUUUAGGUAA
749-755 of JARID2 3´ UTR	5´.......................GGAAAUCCAUU
hsa-miR-1246	3´-GGACGAGGUUUUUAGGUAA

hsa-miR-432-5p

16-23 of HMG20A 3´ UTR	5´.......................GAACUCCAAGA
hsa-miR-432-5p	3´-GGUGGGUUACUGGAUGAGGUUCU
705-711 of ZNF417 3´ UTR	5´.......................UCACUCCAAGC
hsa-miR-432-5p	3´-GGUGGGUUACUGGAUGAGGUUCU
4505-4511 of ZNF417 3´ UTR	5´.......................UCUCUCCAAGC
hsa-miR-432-5p	3´-GGUGGGUUACUGGAUGAGGUUCU

hsa-miR-3195

324-330 of ZNF282 3´ UTR	5´.......................GCCCCGGCGCC
hsa-miR-3195	3´-UUGGGCCCGGGCCGCGC
4206-4212 of HIF1A 3´ UTR	5´.......................CGCCCGGCGCG
hsa-miR-3195	3´-UUGGGCCCGGGCCGCGC
859-865 of AIP 3´ UTR	5´.......................CCCCCGGCGCU
hsa-miR-3195	3´-UUGGGCCCGGGCCGCGC

hsa-miR-320a

252-259 of ETFA 3´ UTR	5´.......................GCCCAGCUUUA
hsa-miR-320a	3´-AGCGGGAGAGUUGGGUCGAAAA
265-272 of TRIAP1 3´ UTR	5´.......................AAUCAGCUUUA
hsa-miR-320a	3´-AGCGGGAGAGUUGGGUCGAAAA
10554-10560 of NABP1 3´ UTR	5´.......................UUGUAGCUUUA
hsa-miR-320a	3´-AGCGGGAGAGUUGGGUCGAAAA

hsa-miR-320c

424-431 of DAZAP1 3´ UTR	5´.......................CUUCAGCUUUA
hsa-miR-320c	3´-UGGGAGAGUUGGGUCGAAAA
1930-1937 of ARL8B 3´ UTR	5´.......................UCUCAGCUUUA
hsa-miR-320c	3´-UGGGAGAGUUGGGUCGAAAA
252-259 of ETFA 3´ UTR	5´.......................CCACAGCUUUA
hsa-miR-320c	3´-UGGGAGAGUUGGGUCGAAAA

hsa-miR-935

290-297 of UFC1 3´ UTR	5´.......................GGGGUAACUGA
hsa-miR-935	3´-CGCCAUCGCCUUCGCCAUUGACC
138-145 of PMCH 3´ UTR	5´.......................UCAGUAACUGA
hsa-miR-935	3´-CGCCAUCGCCUUCGCCAUUGACC
197-204 of HDHD2 3´ UTR	5´.......................UUUGUAACUGA
hsa-miR-935	3´-CGCCAUCGCCUUCGCCAUUGACC

hsa-miR-193a-3p

1228-1235 of PCDHA5 3´UTR	5´................GCGAAGACCCA
hsa-miR-193a-3p	3´-AGUAGAGCGGGCGUUUCUGGGU
248-255 of CA7 3´UTR	5´................UCCAAGACCCA
hsa-miR-193a-3p	3´-AGUAGAGCGGGCGUUUCUGGGU
471-478 of ACVR1 3´UTR	5´................UUAAAGACCCA
hsa-miR-193a-3p	3´-AGUAGAGCGGGCGUUUCUGGGU

hsa-miR-3175

7360-7367 of ZNF652 3´UTR	5´................UUGUCUCCCCA
hsa-miR-3175	3´-UGCAGUGACGCAAGAGAGGGGC
892-898 of MFSD12 3´UTR	5´................CCCCCUCCCCA
hsa-miR-3175	3´-UGCAGUGACGCAAGAGAGGGGC
1181-1187 of CYB5D2 3´UTR	5´................ACCUCUCCCCG
hsa-miR-3175	3´-UGCAGUGACGCAAGAGAGGGGC

hsa-miR-107

126-133 of PPP6R2 3´UTR	5´................UAAAUGCUGCA
hsa-miR-107	3´-ACUAUCGGGACAUGUUACGACGA
775-782 of TRIAP1 3´UTR	5´................AAAAUGCUGCA
hsa-miR-107	3´-ACUAUCGGGACAUGUUACGACGA
2516-2522 of ANO3 3´UTR	5´................UGAAUGCUGCU
hsa-miR-107	3´-ACUAUCGGGACAUGUUACGACGA

hsa-miR-4443

67-74 of IMMP2L 3´UTR	5´................GAAGCCUCCAA
hsa-miR-4443	3´-UUUUGGGUGCGGAGGUU
694-701 of SLC30A2 3´UTR	5´................ACAGCCUCCAA
hsa-miR-4443	3´-UUUUGGGUGCGGAGGUU
148-155 of MOGAT3 3´UTR	5´................CAUGCCUCCAA
hsa-miR-4443	3´-UUUUGGGUGCGGAGGUU

hsa-miR-15b-5p

1568-1575 of PLSCR4 3´UTR	5´................ACAUGCUGCUA
hsa-miR-15b-5p	3´-ACAUUUGGUACUACACGACGAU
1771-1778 of WNT3A 3´UTR	5´................UGAUGCUGCUA
hsa-miR-15b-5p	3´-ACAUUUGGUACUACACGACGAU
246-253 of TMEM74B 3´UTR	5´................UUCUGCUGCUA
hsa-miR-15b-5p	3´-ACAUUUGGUACUACACGACGAU

hsa-miR-151a-5p

928-935 of KIAA1429 3´UTR	5´................AUACUCCUCGA
hsa-miR-151a-5p	3´-UGAUCUGACACUCGAGGAGCU
4077-4083 of N4BP1 3´UTR	5´................GAGCUCCUCGU
hsa-miR-151a-5p	3´-UGAUCUGACACUCGAGGAGCU
102-109 of RALGAPA1 3´UTR	5´................UGUCUCCUCGU
hsa-miR-151a-5p	3´-UGAUCUGACACUCGAGGAGCU

hsa-miR-210-3p

250-257 of ISCU 3´UTR	5´................GAUACGCACAA
hsa-miR-210-3p	3´-AGUCGGCGACAGUGUGCGUGUC
250-257 of DIMT1 3´UTR	5´................GAGACGCACAA
hsa-miR-210-3p	3´-AGUCGGCGACAGUGUGCGUGUC
1832-1839 of FAM73B 3´UTR	5´................UCAACGCACAA
hsa-miR-210-3p	3´-AGUCGGCGACAGUGUGCGUGUC

hsa-miR-3687

61-74 of SIRT2 3´UTR	5´................CCUUGUCCGGA
hsa-miR-3687	3´-UGCAGCGUGCUUGCGGACAGGCCC
254-261 of DDX46 3´UTR	5´................AAAUGUCCGGA
hsa-miR-3687	3´-UGCAGCGUGCUUGCGGACAGGCCC
278-285 of TFG 3´UTR	5´................CCUUGUCCGGA
hsa-miR-3687	3´-UGCAGCGUGCUUGCGGACAGGCCC

hsa-miR-4485

34-41 of PARK7 3´UTR	5´................ACAGGCCGUUA
hsa-miR-4485	3´-AAUCCCAUGGCCCCGGCAAU
216-223 of GALNT14 3´UTR	5´................CAAGGCCGUUA
hsa-miR-4485	3´-AAUCCCAUGGCCCCGGCAAU
591-598 of PPAPDC3 3´UTR	5´................AAAGGCCGUUA
hsa-miR-4485	3´-AAUCCCAUGGCCCCGGCAAU

hsa-miR-106a-5p

107-114 of HN1 3´UTR	5´................GAAGCACUUUA
hsa-miR-106a-5p	3´-GAUGGACGUGACAUUCGUGAAAA
114-121 of NAGK 3´UTR	5´................AAUGCACUUUA
hsa-miR-106a-5p	3´-GAUGGACGUGACAUUCGUGAAAA
27-34 of HAUS8 3´UTR	5´................UGAGCACUUUA
hsa-miR-106a-5p	3´-GAUGGACGUGACAUUCGUGAAAA

hsa-miR-1275

103-110 of SPRR1B 3´UTR	5´................GUCUCCCCCAA
hsa-miR-1275	3´-CUGUCGGAGAGGGGGUG
26-33 of TKT4 3´UTR	5´................CAGUCCCCCAA
hsa-miR-1275	3´-CUGUCGGAGAGGGGGUG
241-248 of NTN5 3´UTR	5´................GUUUCCCCCAA
hsa-miR-1275	3´-CUGUCGGAGAGGGGGUG

hsa-miR-7150

989-985 of ZNF512B 3´UTR	5´................UGUCUGCCAA
hsa-miR-7150	3´-AUGGAGAGGGGGACGGUC
154-161 of LTV1 3´UTR	5´................UCCCCUGCCAA
hsa-miR-7150	3´-AUGGAGAGGGGGACGGUC
1435-1442 of NSG2 3´UTR	5´................UAUCCUGCCAA
hsa-miR-7150	3´-AUGGAGAGGGGGACGGUC

hsa-miR-7977

3413-3419 of MUC19 3´UTR	5´................UCAGCUGGGAU
hsa-miR-7977	3´-ACCACGCAACCGACCCUU
36-43 of WFDC11 3´UTR	5´................UGGGCUGGGAA
hsa-miR-7977	3´-ACCACGCAACCGACCCUU
65-72 of OR2D2 3´UTR	5´................CUUGCUGGGAA
hsa-miR-7977	3´-ACCACGCAACCGACCCUU

hsa-miR-4656

38-45 of NYP 3´UTR	5´................UUUUCAGCCCA
hsa-miR-4656	3´-UGUCCGGAGGACGGGAGUCGGGU
56-63 of MIA 3´UTR	5´................CAAUCAGCCCA
hsa-miR-4656	3´-UGUCCGGAGGACGGGAGUCGGGU
3039-3045 of FBXO41 3´UTR	5´................UGCCCAGCCCA
hsa-miR-4656	3´-UGUCCGGAGGACGGGAGUCGGGU

hsa-miR-504-3p

353-360 of MTRNR2L5 3´UTR	5´................ACUGCACUCCA
hsa-miR-504-3p	3´-CUUUGGGACGGGACGUGAGGG
18-25 of RP11-81K2.1 3´UTR	5´................AUUGCACUCCA
hsa-miR-504-3p	3´-CUUUGGGACGGGACGUGAGGG
26-33 of IFITM1 3´UTR	5´................UUUGCACUCCA
hsa-miR-504-3p	3´-CUUUGGGACGGGACGUGAGGG

hsa-miR-6781-5p

109-116 of CNBD2 3´UTR	5´................AAACCGGCCCA
hsa-miR-6781-5p	3´-UGCGGGAACUGGAGGCCGGGC
20-27 of LCN12 3´UTR	5´................UGUCCGGCCCA
hsa-miR-6781-5p	3´-UGCGGGAACUGGAGGCCGGGC
59-66 of DUX4L2 3´UTR	5´................GAGCCGGCCCA
hsa-miR-6781-5p	3´-UGCGGGAACUGGAGGCCGGGC

Figure S1. Shows representative examples of mRNA targets for the 31 DEmiRNAs, as TargetScan and miRTargetLink2.0 software's predicted.

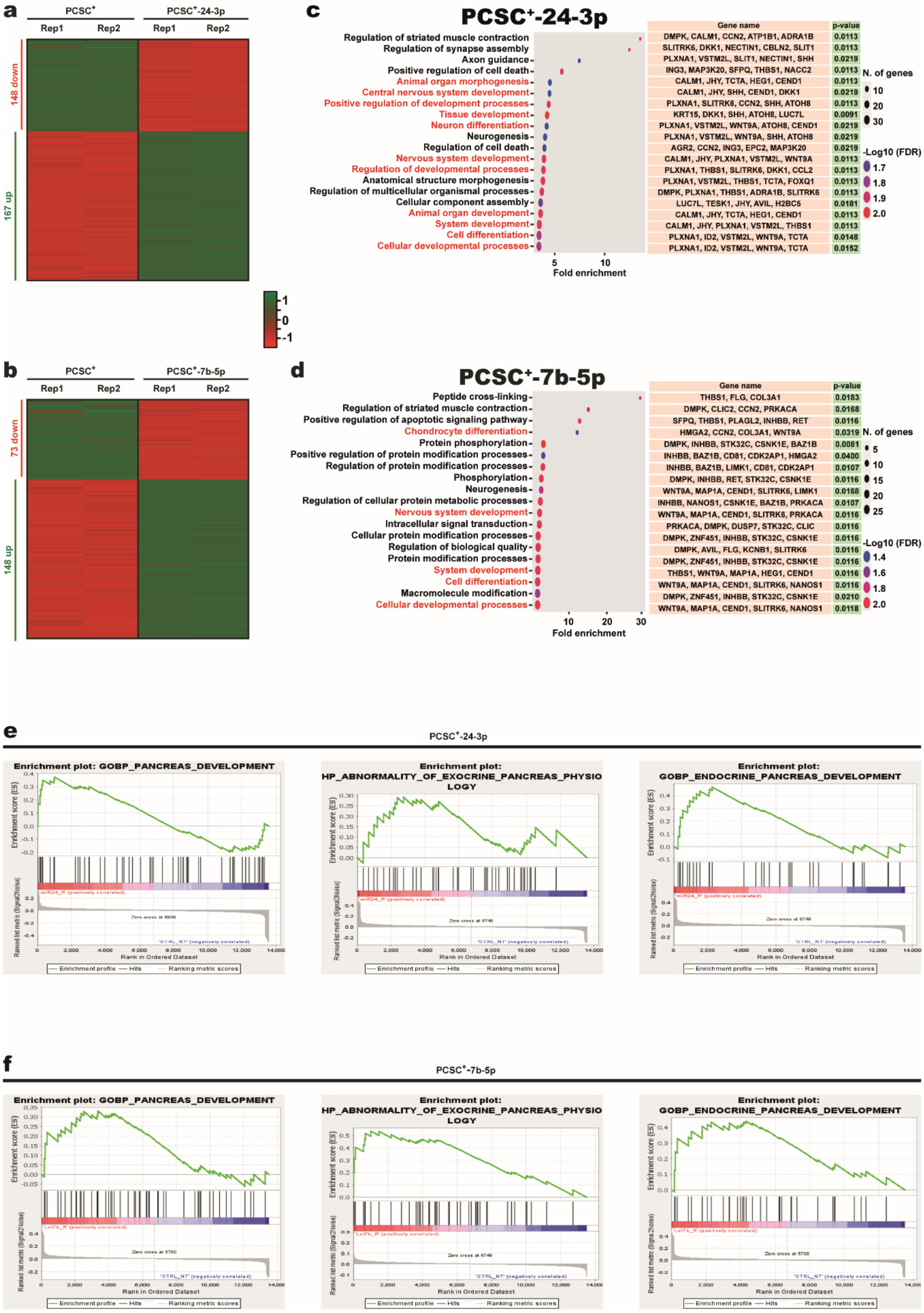
a
PCSC+
PCSC+-24-3p
Rep1
Rep2
Rep1
Rep2
148 down
167 up
1
0
-1
b
PCSC+
PCSC+-7b-5p
Rep1
Rep2
Rep1
Rep2
73 down
148 up
c
PCSC+-24-3p
Regulation of striated muscle contraction
Regulation of synapse assembly
Axon guidance
Positive regulation of cell death
Animal organ morphogenesis
Central nervous system development
Positive regulation of development processes
Tissue development
Neuron differentiation
Neurogenesis
Regulation of cell death
Nervous system development
Regulation of developmental processes
Anatomical structure morphogenesis
Regulation of multicellular organismal processes
Cellular component assembly
Animal organ development
System development
Cell differentiation
Cellular developmental processes
5
10
Fold enrichment
Gene name | p-value
DMPK, CALM1, CCN2, ATP1B1, ADRA1B | 0.0113
SLITRK6, DKK1, NECTIN1, CBLN2, SLIT1 | 0.0113
PLXNA1, VSTM2L, SLIT1, NECTIN1, SHH | 0.0219
ING3, MAP3K20, SFPQ, THBS1, NACC2 | 0.0113
CALM1, JHY, TCTA, HEG1, CEND1 | 0.0113
CALM1, JHY, SHH, CEND1, DKK1 | 0.0219
PLXNA1, SLITRK6, CCN2, SHH, ATOH8 | 0.0113
KRT15, DKK1, SHH, ATOH8, LUC7L | 0.0091
PLXNA1, VSTM2L, WNT9A, ATOH8, CEND1 | 0.0219
PLXNA1, VSTM2L, WNT9A, SHH, ATOH8 | 0.0219
AGR2, CCN2, ING3, EPC2, MAP3K20 | 0.0219
CALM1, JHY, PLXNA1, VSTM2L, WNT9A | 0.0113
PLXNA1, THBS1, SLITRK6, DKK1, CCL2 | 0.0113
PLXNA1, VSTM2L, THBS1, TCTA, FOXQ1 | 0.0113
DMPK, PLXNA1, THBS1, ADRA1B, SLITRK6 | 0.0113
LUC7L, TESK1, JHY, AVIL, H2BC5 | 0.0181
CALM1, JHY, TCTA, HEG1, CEND1 | 0.0113
CALM1, JHY, PLXNA1, VSTM2L, THBS1 | 0.0113
PLXNA1, ID2, VSTM2L, WNT9A, TCTA | 0.0148
PLXNA1, ID2, VSTM2L, WNT9A, TCTA | 0.0152
N. of genes
10
20
30
-Log10 (FDR)
1.7
1.8
1.9
2.0
d
PCSC+-7b-5p
Peptide cross-linking
Regulation of striated muscle contraction
Positive regulation of apoptotic signaling pathway
Chondrocyte differentiation
Protein phosphorylation
Positive regulation of protein modification processes
Regulation of protein modification processes
Phosphorylation
Neurogenesis
Regulation of cellular protein metabolic processes
Nervous system development
Intracellular signal transduction
Cellular protein modification processes
Regulation of biological quality
Protein modification processes
System development
Cell differentiation
Macromolecule modification
Cellular developmental processes
10
20
30
Fold enrichment
Gene name | p-value
THBS1, FLG, COL3A1 | 0.0183
DMPK, CLIC2, CCN2, PRKACA | 0.0168
SFPQ, THBS1, PLAGL2, INHBB, RET | 0.0116
HMGA2, CCN2, COL3A1, WNT9A | 0.0319
DMPK, INHBB, STK32C, CSNK1E, BAZ1B | 0.0081
INHBB, BAZ1B, CD81, CDK2AP1, HMGA2 | 0.0400
INHBB, BAZ1B, LIMK1, CD81, CDK2AP1 | 0.0107
DMPK, INHBB, RET, STK32C, CSNK1E | 0.0116
WNT9A, MAP1A, CEND1, SLITRK6, LIMK1 | 0.0168
INHBB, NANOS1, CSNK1E, BAZ1B, PRKACA | 0.0107
WNT9A, MAP1A, CEND1, SLITRK6, PRKACA | 0.0116
PRKACA, DMPK, DUSP7, STK32C, CLIC | 0.0116
DMPK, ZNF451, INHBB, STK32C, CSNK1E | 0.0116
DMPK, AVIL, FLG, KCNB1, SLITRK6 | 0.0116
DMPK, ZNF451, INHBB, STK32C, CSNK1E | 0.0116
THBS1, WNT9A, MAP1A, HEG1, CEND1 | 0.0116
WNT9A, MAP1A, CEND1, SLITRK6, NANOS1 | 0.0116
DMPK, ZNF451, INHBB, STK32C, CSNK1E | 0.0210
WNT9A, MAP1A, CEND1, SLITRK6, NANOS1 | 0.0118
N. of genes
5
10
15
20
25
-Log10 (FDR)
1.4
1.6
1.8
2.0
e
PCSC+-24-3p
Enrichment plot: GOBP_PANCREAS_DEVELOPMENT
Enrichment plot: HP_ABNORMALITY_OF_EXOCRINE_PANCREAS_PHYSIOLOGY
Enrichment plot: GOBP_ENDOCRINE_PANCREAS_DEVELOPMENT
Enrichment score (ES)
Ranked list metric (Signal2Noise)
Rank in Ordered Dataset
Enrichment profile
Hits
Ranking metric scores
f
PCSC+-7b-5p
Enrichment plot: GOBP_PANCREAS_DEVELOPMENT
Enrichment plot: HP_ABNORMALITY_OF_EXOCRINE_PANCREAS_PHYSIOLOGY
Enrichment plot: GOBP_ENDOCRINE_PANCREAS_DEVELOPMENT
Enrichment score (ES)
Ranked list metric (Signal2Noise)
Rank in Ordered Dataset
Enrichment profile
Hits
Ranking metric scores

Figure S2. Transcriptomic analysis of PCSC+ cells overexpressing miR-24-3p or let-7b-5p. (a, b) Heatmaps of DEGs in PCSC+ cells transfected with miR-24-3p or let-7b-5p mimics. DEGs were identified with a FDR < 0.05. Upregulated genes are shown in green; downregulated genes in red.
(c, d) Biological processes enriched in transfected cells identified via ShinyGO, with representative genes listed. **(e, f)** Gene Set Enrichment Analysis (GSEA) reveals enrichment of pancreatic differentiation-related pathways (GOBP_PANCREAS_DEVELOPMENT, GOBP_ENDOCRINE_PANCREAS_DEVELOPMENT, HP_ABNORMALITY_OF_EXOCRINE_PANCREAS_PHYSIOLOGY) in miRNA-overexpressing PCSC+ cells versus controls. Significance thresholds: FDR = 0.05; p = 0.05.

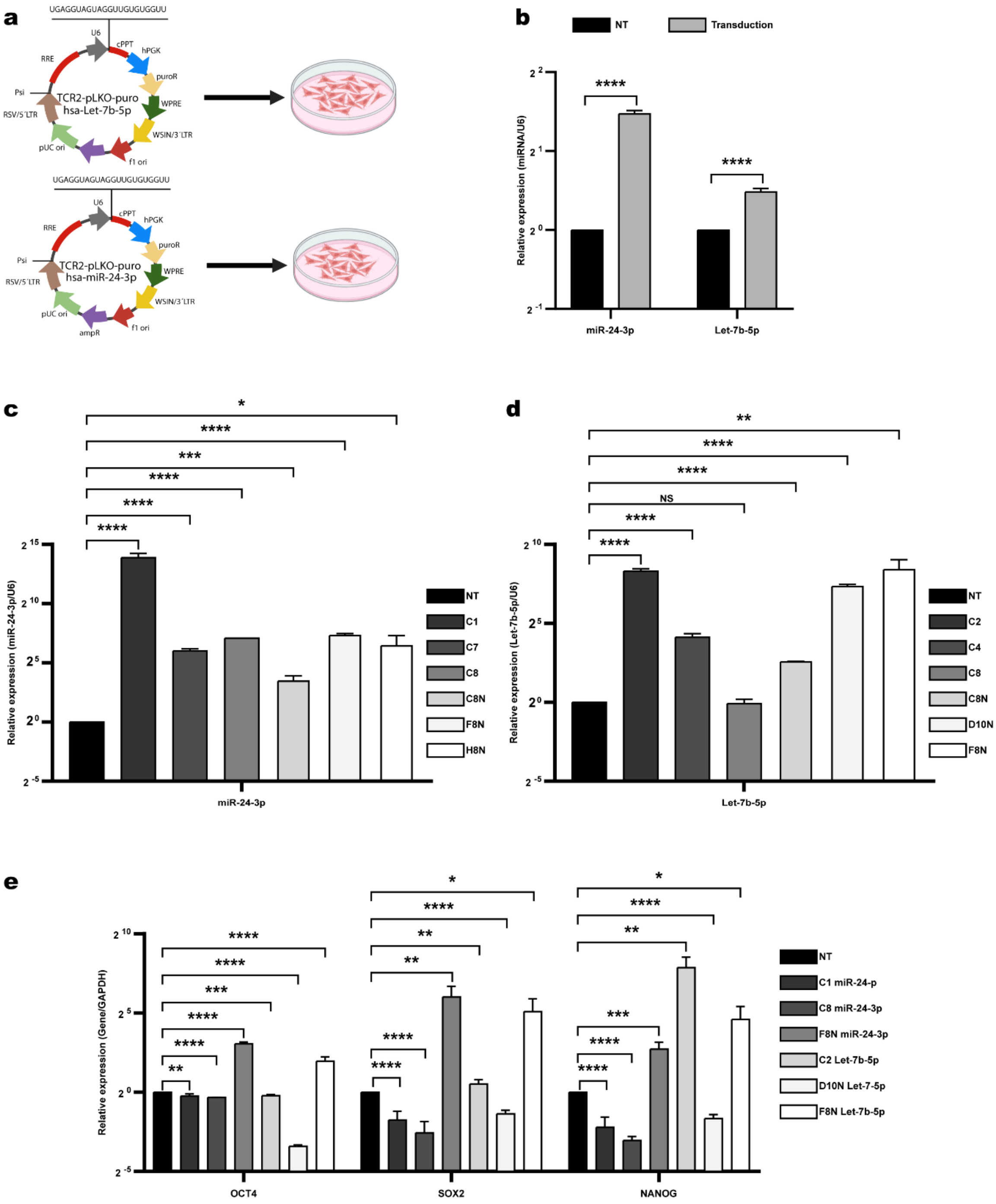

Figure S3. Isolation and characterization of PCSC clones overexpressing let-7b-5p or miR-24-3p. (a) PCSCs were transduced with lentiviral particles expressing human miR-24-3p or let-7b-5p; non-transduced PCSCs served as controls. **(b)** Relative miRNA expression in transduced cells compared to controls, showing significant upregulation (**** $p < 0.0001$, two-tailed Student's *t*-test).

(c, d) RT-qPCR confirms variable overexpression levels of miR-24-3p and let-7b-5p across seven isolated clones. Statistical significance indicated (NS: not significant; * $p < 0.05$; ** $p < 0.01$; *** $p < 0.001$; **** $p < 0.0001$).
(e) Overexpression of miR-24-3p or let-7b-5p significantly downregulates pluripotency factor expression relative to GAPDH (two-tailed Student's *t*-test). Statistical significance: * $p < 0.05$; ** $p < 0.01$; *** $p < 0.001$; **** $p < 0.0001$.
